# Mathematical modeling of cardiac function to evaluate clinical cases in adults and children

**DOI:** 10.1371/journal.pone.0224663

**Published:** 2019-10-31

**Authors:** Selim Bozkurt

**Affiliations:** Institute of Cardiovascular Science, University College London, London, United Kingdom; University of Minnesota, UNITED STATES

## Abstract

Time-varying elastance models can simulate only the pressure and volume signals in the heart chambers while the diagnosis of clinical cases and evaluation of different treatment techniques require more information. In this study, an extended model utilizing the geometric dimensions of the heart chambers was developed to describe the cardiac function. The new cardiac model was evaluated by simulating a healthy and dilated cardiomyopathy (DCM) condition for adults and children. The left ventricular ejection fraction, end-diastolic volume, end-diastolic diameter and diastolic sphericity index were 53.60%, 125 mL, 5.08 cm and 1.82 in the healthy adult cardiovascular system model and 23.70%, 173 mL, 6.60 cm and 1.40 in the DCM adult cardiovascular system model. In the healthy child cardiovascular system model, the left ventricular ejection fraction, end-diastolic volume, end-diastolic diameter and diastolic sphericity index were 59.70%, 92 mL, 4.10 cm and 2.26 respectively and 30.70%, 125 mL, 4.94 cm and 1.87 in the DCM child cardiovascular system model. The developed cardiovascular system model simulates the hemodynamic variables and clinical diagnostic indicators within the physiological range for healthy and DCM conditions proving the feasibility of this new model to evaluate clinical cases in adults and children.

## Introduction

Numerical modeling of cardiac physiology plays an important role to evaluate clinical scenarios and outcomes of different treatment techniques before experimental or clinical applications. Nonetheless, the information obtained from a numerical simulation depends on the modeled relations in the utilized model. For instance, time-varying elastance models describe the pressure-volume relations in the heart chambers using an elastance function which changes with respect to time over a cardiac cycle [1]. The time-varying elastance models have been used for different clinical scenarios such as evaluating heart failure [2], left ventricular assist device support [3] or simulating the interaction between the cardiovascular and respiratory systems [4,5]. Although time-varying elastance models are widely used to model the cardiac function, they simulate only the blood pressure and volume in heart chambers.

More detailed numerical models such as single fiber contraction models describe the cardiac function by simulating the contraction of a sarcomere over a cardiac cycle [6]. The single fiber contraction models allow simulating the fiber tension in a heart chamber, in addition to the blood pressure and volume [7]. The single fiber contraction models have also been used to simulate physiological and clinical scenarios such as the influence of intra-myocardial pressure on the coronary arterial blood flow rate [8], simulation of fetal heart rate variability [9] or evaluation of rotary blood pump support [10] etc. Although these models are driven by the fiber contraction and provide more information about the cardiac physiology, they also remain insufficient to simulate different mechanisms occurring at each level of cardiac contraction and do not provide information about clinical indicators such as heart chamber dimensions for the physiological cases.

Multi-scale models of the cardiac dynamics simulating the physiological processes at the cellular, protein and organ levels also were developed and used to understand the pathophysiology of heart failure [11]. However, increasing the complexity of a numerical model may not improve the outcome of a simulation. As shown in [12] relatively simple models describing the cardiac function, simulate the physiological and clinical cases such as heart failure and mechanical circulatory support more accurately with respect to the multiscale models because the small discrepancies between the real physiology and the numerical model in each scale cause large deviations at the organ level. Moreover, lumped parameter modeling mostly was used to simulate the adult cardiovascular system. Numerical simulation of cardiac function in children remains to work further to evaluate different physiological scenarios as only a small number of studies focus on pediatric cases [13]. A detailed review of lumped parameter models is given in [14].

The similarities and differences between children and adult physiologies have been subject to developmental theories [15]. Hemodynamic parameters and size of the heart in children reaches similar values of the adult cardiovascular system in time. Therefore, a numerical model capable of simulating the dimensions of the heart chambers and related parameters used to diagnose clinical cases and describe the cardiac function not only for adults but also for children can be utilized to evaluate clinical cases and effects of different treatment in pediatric patients at different ages.

The aim of this study is to develop a lumped parameter model describing the cardiac function and circulatory system and simulating the diagnostic criteria for the clinical cases, therefore, can be used to evaluate physiological scenarios and treatment techniques for cardiovascular system diseases in adults and children at different ages.

## Methods

The mathematical model proposed in this study describes the relationship between the pressure and radius in the heart chambers. Left ventricular geometry resembles a truncated ellipsoid [16], therefore, an ellipsoidal geometry was used for the modeling of the left ventricle. The left ventricular volume (*V*_*lv*_) was expressed using the left ventricular radius (*r*_*lv*_), long axis length (*l*_*lv*_) and an additional coefficient (*K*_*lv*_) which allows to include effects of the contraction in the long axis and scales the proportion between the left ventricular radius and volume over a cardiac cycle.

$$V_{lv}=\frac{(4/3)\pi K_{lv}r_{lv}^{2}l_{lv}}{2}$$ (1)

Ventricular long axis length (*l*_*lv*_) assumed to be constant over a cardiac cycle, so, the left ventricular radius (*r*_*lv*_) and the change of the left ventricular radius with respect to time (*dr*_*lv*_*/dt*) can be described as given below.

$$r_{lv}={\left(\frac{6V_{lv}}{4\pi K_{lv}l_{lv}}\right)}^{1/2}$$ (2)

$$\frac{dr_{lv}}{dt}=\frac{3\left(dV_{lv}/dt\right)}{4\pi K_{lv}l_{lv}}{\left(\frac{6V_{lv}}{4\pi K_{lv}l_{lv}}\right)}^{-1/2}$$ (3)

Change of the left ventricular volume with respect to time (*dV*_*lv*_*/dt*) can also be described as the difference between the mitral and aortic valve flow rates (*Q*_*mv*_, *Q*_*av*_).

$$\frac{dV_{lv}}{dt}=Q_{mv}-Q_{av}$$ (4)

Change of the left ventricular radius with respect to time (*dr*_*lv*_*/dt*) becomes as given below.

$$\frac{dr_{lv}}{dt}=\frac{3\left(Q_{mv}-Q_{av}\right)}{4\pi K_{lv}l_{lv}}{\left(\frac{6V_{lv}}{4\pi K_{lv}l_{lv}}\right)}^{-1/2}$$ (5)

The left ventricular pressure during the active contraction and the relaxation phases (*p*_*lv*,*a*_) is described using an activation function (*f*_*act*,*lv*_), end-systolic elastance (*E*_*es*,*lv*_), the left ventricular radius (*r*_*lv*_) and left ventricular radius at zero pressure volume (*r*_*lv*,*0*_).

$$p_{lv,a}(t)=E_{es,lv}\left[\frac{4}{6}\pi Kl_{lv}\left(r_{lv}^{2}-r_{lv,0}^{2}\right)\right]f_{act,lv}(t)$$ (6)

Here, the expression in the square brackets shows the difference between the left ventricular volume (*V*_*lv*_) as described in the Eq 1 and the left ventricular zero pressure-volume (*V*_*lv*,*0*_). The activation function (*f*_*act*,*lv*_) driving the left ventricular contraction is adapted from [17] and given below.

$$f_{act,lv}(t)=\{\begin{array}{ll} \frac{1-\text{cos}\left((t/T_{1})\pi \right)\;}{2} & 0\leq t<T_{1}\\ \frac{1+\text{cos}\left((t-T_{1})/(T_{2}-T_{1})\pi \right)\;}{2} & T_{1}\leq t<T_{2}\\ 0 & T_{2}\leq t<T \end{array}$$ (7)

Here, *t* is the time over a cardiac cycle, *T*_*1*_, *T*_*2*,_ and *T* are the times at the end of systole, end of the ventricular relaxation and duration of the cardiac cycle.

The relationship between the left ventricular pressure and the radius for the passive left ventricular contraction is adapted from [13] using the Eq 1 as the left ventricular volume.

$$p_{lv,p}(t)=Ae^{\left(B\frac{4}{6}\pi K{\left(r_{lv}(t)\right)}^{2}l_{lv}\right)}-1$$ (8)

The left ventricular pressure (*p*_*lv*_) signal over a cardiac cycle is obtained using active and passive components of the left ventricular pressure (*p*_*lv*,*a*_, *p*_*lv*,*p*_).

$$p_{lv}=p_{lv,a}+p_{lv,p}$$ (9)

Right ventricle has a more complex shape with respect to left ventricle and resembles a crescentic cross-section and a triangle from the side view [18]. The crescentic cross-section of the right ventricle is more extensive with respect to the circular cross-section of the left ventricle, and the triangular side view of right ventricle seems like an ellipsoid which has a larger volume occupied by left ventricle [19]. Therefore, an ellipsoidal volume trimmed at the long axis and the basal axis of a right ventricle provides a quite good approximation to model the right ventricular volume (*V*_*rv*_).

$$V_{rv}=\frac{(4/3)\pi K_{rv}r_{rv}^{2}l_{rv}}{4}$$ (10)

In the equation above, *K*_*rv*_, *l*_*rv*_ and *r*_*rv*_ are the scaling coefficient, the right ventricular length from base-to-apex and right ventricular radius. The right ventricular radius (*r*_*rv*_) and the change of the right ventricular radius with respect to time (*dr*_*rv*_*/dt*) are described as given below.

$$r_{rv}={\left(\frac{3V_{rv}}{\pi K_{rv}l_{rv}}\right)}^{1/2}$$ (11)

$$\frac{dr_{rv}}{dt}=\frac{3\left(Q_{tv}-Q_{pv}\right)}{2\pi K_{rv}l_{rv}}{\left(\frac{3V_{rv}}{\pi K_{rv}l_{rv}}\right)}^{-1/2}$$ (12)

In the equations above, *Q*_*tv*_ and *Q*_*pv*_ are the flow rates through the tricuspid and pulmonary valves. The right ventricular active and passive pressure components (*p*_*rv*,*a*_, *p*_*rv*,*p*_) are modeled as given below. The right ventricular pressure is obtained using active and passive pressure components.

$$p_{rv,a}(t)=E_{es,rv}\left[\frac{1}{3}\pi Kl_{rv}\left(r_{rv}^{2}-r_{rv,0}^{2}\right)\right]f_{act,rv}(t)$$ (13)

$$p_{rv,p}(t)=Ae^{\left(B\frac{1}{3}\pi K{\left(r_{rv}(t)\right)}^{2}l_{rv}\right)}-1$$ (14)

The left and right atrial geometries were modeled using a similar model to the left ventricular geometry. Therefore, the same relations for the left and right atrial volumes and radiuses are used as in the left ventricle model with different parameter values. The atrial pressure-radius relationship is modeled adopting a time-varying elastance pressure-volume and expressing the atrial volume as a truncated ellipsoidal shape.

$$p_{la}(t)=E_{la}(t)\left[\frac{4}{6}\pi K_{la}l_{la}\left(r_{la}^{2}-r_{la,0}^{2}\right)\right]$$ (15)

Here, *p*_*la*_ is the left atrial pressure, *K*_*la*_, *l*_*la*_, *r*_*la*_ and *r*_*la*,*0*_ are the left atrial scaling coefficient, length, radius and radius at zero pressure. *E*_*la*_ is the left atrial elastance function and is described using the left atrial activation function (*f*_*act*,*la*_) as given below.

$$E_{la}(t)=E_{\text{min},la}+0.5\left(E_{\text{max},la}-E_{\text{min},la}\right)f_{act.la}(t-D)$$ (16)

$$f_{act,la}(t)=\{\begin{array}{ll} 0 & 0\leq t<T_{a}\\ 1-\text{cos}\left(2\pi \frac{t-T_{a}}{T-T_{a}}\right) & T_{a}\leq t<T \end{array}$$ (17)

The right atrial pressure-radius relations are described in the same way as the left atrial pressure-radius relations. The parameter values used in the adult atria and ventricle models are given in Table 1.

**Table 1:** The parameter values used in the adult atria and ventricle models, the parameter values used in the simulation of DCM are given in the brackets.

	Left Ventricle	Right Ventricle	Left Atrium	Right Atrium
l [cm]	8	8	5.5	5.5
K	1.15 (0.95)	1.75	1.20	1.20
V_0_ [mL]	15 (25)	40	5	5
E_es_ [mmHg/mL]	2.5 (0.9)	1	-	-
A	1 (0.65)	1	-	-
B	0.02	0.02	-	-
E_max_ [mmHg/mL]	-	-	0.3	0.3
E_min_ [mmHg/mL]	-	-	0.2	0.2
T_1_ [s]	0.33*T	0.33*T	-	-
T_2_ [s]	0.45*T	0.45*T	-	-
T_a_ [s]	-	-	0.8*T	0.8*T
T [s]	0.8	0.8	0.8	0.8
D [s]	-	-	0.04	0.04

The longitudinal lengths of the left and right ventricles and atria were selected considering the anatomical ranges given in [20–23] for the adult cardiovascular system model. *K* values were adjusted to simulate the end-systolic and end-diastolic heart chamber radiuses (*r*) within the reference range for the physiological heart chamber pressures and volumes. The zero pressure volume values of the heart chambers (*V*_*0*_) reported in the literature [17,24] used in the healthy adult cardiovascular system model. The left and right ventricular end-systolic elastance values (*E*_*es*_) were selected as 2.5 mmHg/mL and 1 mmHg/mL similar to the reported values in [25,26]. The coefficients in the passive component of the ventricular pressure signals (*A*, *B*) were 1 and 0.02 [13]. The parameters describing the onset of the ventricular and atrial contraction and relaxation times (*T*_*1*_, *T*_*2*_, *T*_*a*_, *T*, *D*) were selected considering the duration of the cardiac cycle phases [27].

**Fig 1:**
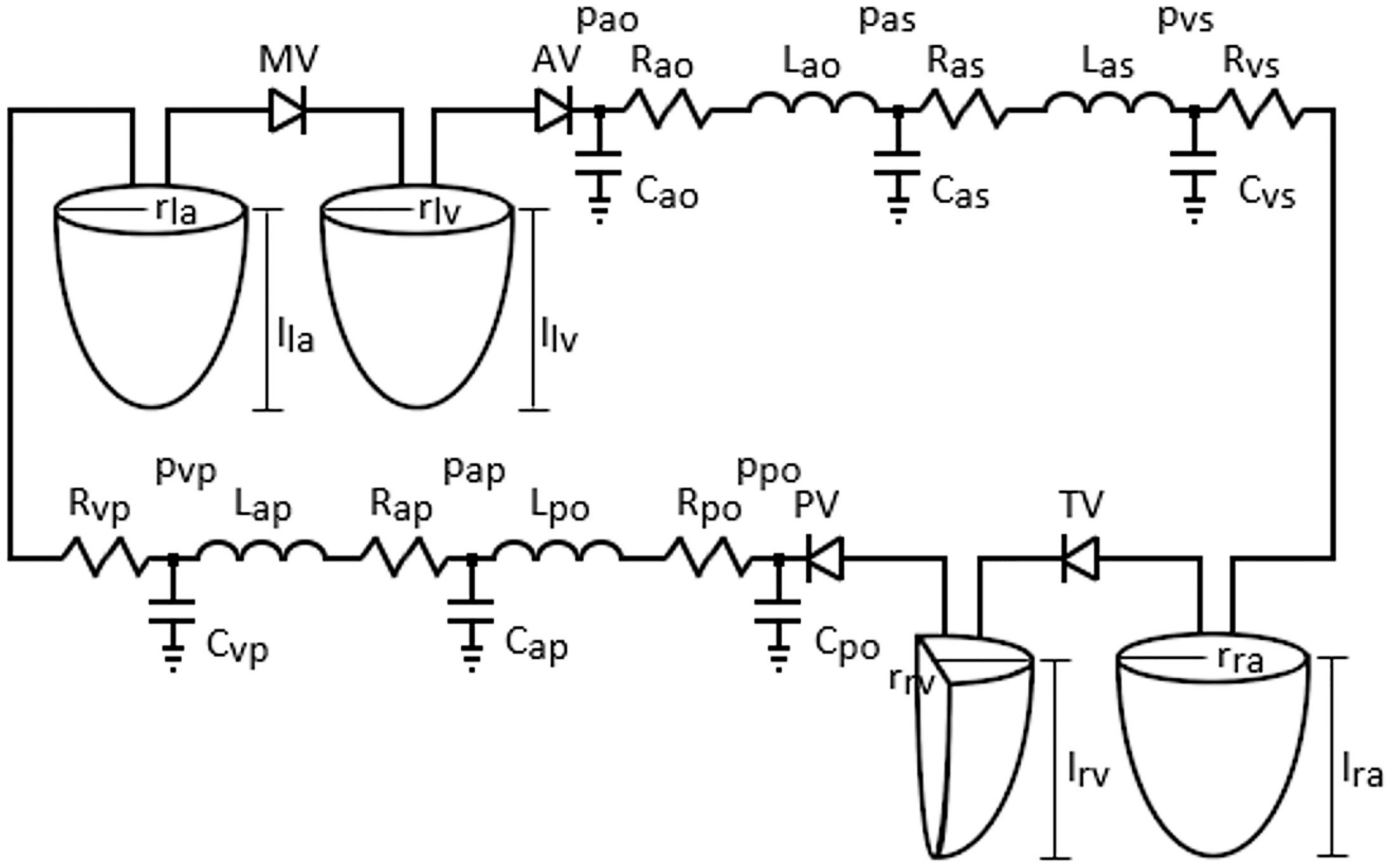
The time-varying geometric models of the heart chambers and electric-analogue of the cardiovascular system. *R*, *L* and *C* denote resistance, inertance and compliance, *p* and denote pressure, *MV*, *AV*, *TV* and *PV* are mitral, aortic, tricuspid and pulmonary valves, *r* and *l* are radius and length, *la*, *lv*, *ra* and *rv* denote left atrium and ventricle and right atrium and ventricle, *ao*, *as*, *vs* denote aorta, systemic arterioles and systemic veins, *po*, *ap*, *vp* pulmonary arteries, pulmonary arterioles and pulmonary veins.

The circulation loop was described using a lumped parameter model which includes electrical analogues for the blood vessel resistance, compliance and inertance. The heart valves were modeled as ideal diodes allowing one-way blood flow. The time-varying geometric models of the heart chambers and electric-analogue of the circulatory loop are given in Fig 1. The resistance, compliance and inertance values in the circulatory system were adjusted manually within the reported physiological range for the healthy adult cardiovascular system [28–30]. The parameter values used in the adult circulatory loop are given in Table 2.

**Table 2:** The parameter values used in the adult circulatory loop, the parameter values used in the simulation of DCM are shown in the brackets.

	R [mmHg s/mL]	L [mmHg s^2^/mL]	C [mL/mmHg]
Aorta	0.05	1e-5	0.2
Systemic Arterioles	0.95 (1.6)	1e-5	1.7
Systemic Veins	0.05	-	30
Pulmonary Arteries	0.01	1e-5	5
Pulmonary Arterioles	0.15	1e-5	0.2
Pulmonary Veins	0.05	-	30
Mitral Valve	0.002	-	-
Aortic Valve	0.002	-	-
Tricuspid Valve	0.001	-	-
Pulmonary Valve	0.001	-	-

The healthy child cardiovascular system model was simulated by modifying the parameters used in the healthy adult cardiovascular system model. The model parameters were adjusted to simulate children with 1 m^2^ body surface area and 8–12 age interval [31]. The systolic elastance values in the child cardiovascular system model were selected, considering the data given in [32]. The ventricular long axis length is taken from [33] for the simulated age and body surface area. The zero pressure volumes of the heart chambers (*V*_*0*_) were decreased in the child cardiovascular system model [34]. The compliance values of the blood vessels (*C*) were reduced in the children as the vessel compliance increases with the increasing body surface area and age [35]. The resistances of the blood vessels (*R*) are kept the same in the child cardiovascular system model as in the adult cardiovascular system model because the difference between the adult vascular resistances and children vascular resistances for the considered ages is not very high and the vascular resistances vary in a very high variation range in children [36]. Duration of a cardiac cycle (*T*) was decreased to 0.75 bpm in the child cardiovascular system model as the heart rate increases with the decreasing age [36]. The total blood volume (*V*_*tot*_) is significantly less in children with relatively small body surface area with respect to the adult cardiovascular system [37]. The total blood volume in the children having 1 m^2^ body surface area is around 2000 mL [37]. The parameter values used in the heart chambers and circulatory loop of the child cardiovascular system model are given in Tables 3 and 4.

**Table 3:** The parameter values used in the child atria and ventricle models; the parameter values used in the simulation of DCM are shown in the brackets.

	Left Ventricle	Right Ventricle	Left Atrium	Right Atrium
l [cm]	7	7	4.5	4.5
K	1.50 (1.40)	3.25	2.50	2.50
V_0_ [mL]	10 (17)	25	3	3
E_es_ [mmHg/mL]	3.5 (1.3)	1.4	-	-
A	1 (0.85)	1	-	-
B	0.02	0.02	-	-
E_max_ [mmHg/mL]	-	-	0.4	0.4
E_min_ [mmHg/mL]	-	-	0.2	0.2
T_1_ [s]	0.33*T	0.33*T	-	-
T_2_ [s]	0.45*T	0.45*T	-	-
T_a_ [s]	-	-	0.8*T	0.8*T
T [s]	0.75	0.75	0.75	0.75
D [s]	-	-	0.0375	0.0375

**Table 4:** The parameter values used in the child circulatory loop, the parameter values used in the simulation of DCM are shown in the brackets.

	R [mmHg s/mL]	L [mmHg s^2^/mL]	C [mL/mmHg]
Aorta	0.05	1e-5	0.13
Systemic Arterioles	0.95 (1.4)	1e-5	1.13
Systemic Veins	0.05	-	19.35
Pulmonary Arteries	0.01	1e-5	3.33
Pulmonary Arterioles	0.15	1e-5	0.13
Pulmonary Veins	0.05	-	19.35
Mitral Valve	0.002	-	-
Aortic Valve	0.002	-	-
Tricuspid Valve	0.001	-	-
Pulmonary Valve	0.001	-	-

DCM is a condition which left ventricle is dilated and weakened due to contractile dysfunction [38]. Diastolic dysfunction may also coexist in patients with ejection fraction less than 45 per cent [39]. The systemic arterial pressure is increased to maintain the perfusion pressure in DCM patients. The contractile dysfunction was simulated in the DCM left ventricle reducing end-systolic elastance of the left ventricle (*E*_*es*,*lv*_) in both adult and child cardiovascular system models. The diastolic dysfunction in the left ventricle was simulated by decreasing the coefficient (*A*) used in the Eq 8. The systemic arteriolar resistance was increased to 1.6 mmHg/mL/s from 0.95 mmHg/mL/s to simulate the increased resistance in the systemic circulation in both adult and child cardiovascular system models. The scaling coefficient (*K*_*lv*_) was also increased to simulate the remodeling in the left ventricular wall and the enlargement of the left ventricular cavity. The modified parameters in the left ventricle and circulatory system for both adult and child cardiovascular system models are given in the Tables 1–4 in the brackets.

Fractional shortening (*fs*) and sphericity index (*si*) were used to evaluate healthy and DCM conditions in the adult and child cardiovascular system models along with the ejection fraction, volumes and dimensions of the heart chambers.

$$fs=\frac{\left(EDD-ESD\right)}{EDD}\times 100$$ (18)

$$si=\frac{l}{D_{mid}}$$ (19)

Here, EDD and ESD are the diameters at the end of diastole and systole, *l* is the length of a heart chamber and *D*_*mid*_ is the diameter of the mid-cavity of the heart chambers. The right ventricular basal diameter was assumed to be the same as the right ventricular radius because of the anatomical geometry of right ventricle. The simulations were performed using Matlab Simulink R2017a. The set of equations was solved using the ode15s solver. The maximum step size was 1e-3 s, relative tolerance was set to 1e-3.

## Results

**Fig 2:**
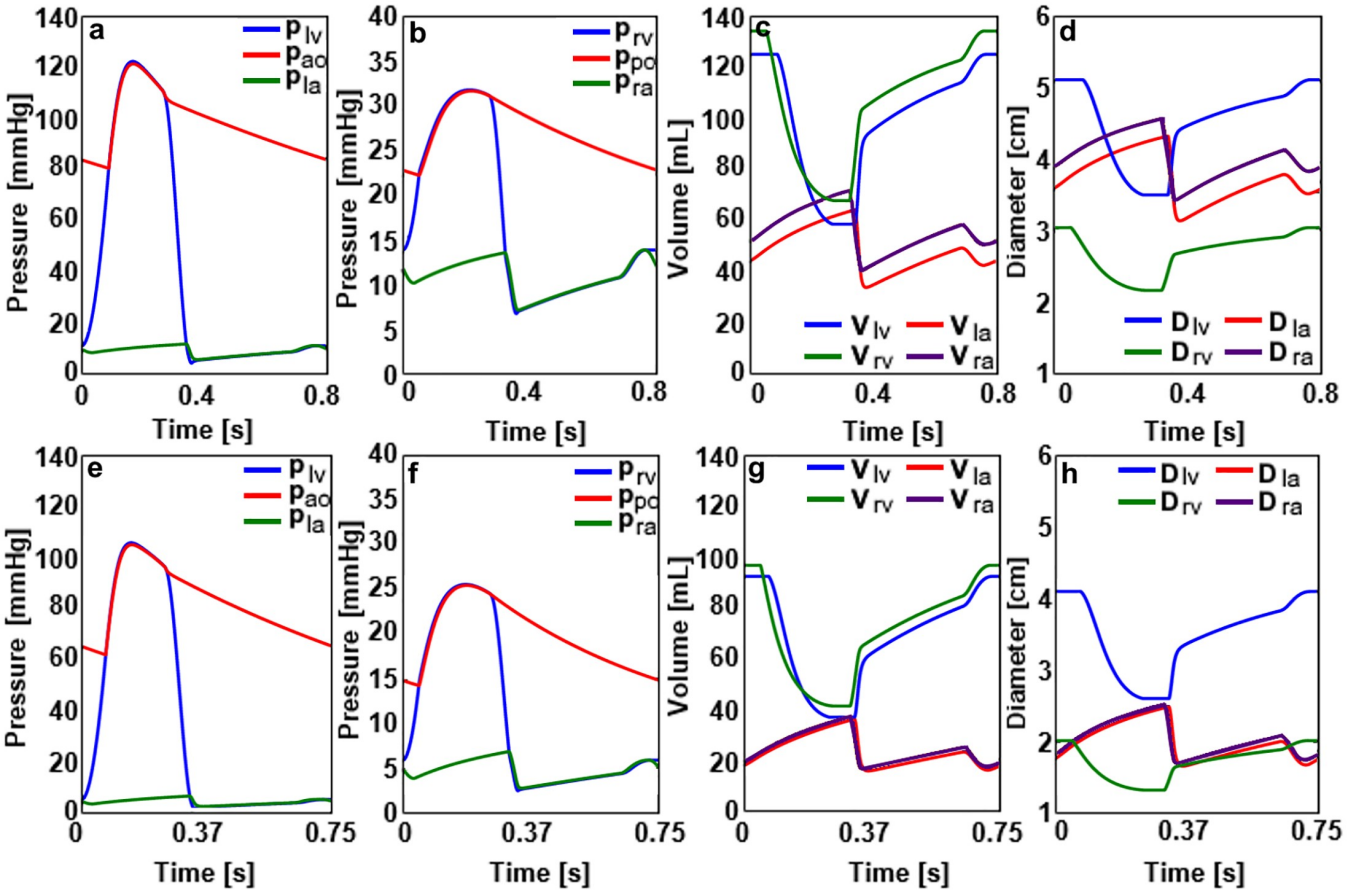
a) The pressure signals in the left ventricle, aorta and left atrium, b) the pressure signals in the right ventricle, pulmonary artery and right atrium, c) the volume signals in the left and right ventricles and atria, d) the ventricular and atrial diameters for the healthy adult cardiovascular system. e) The pressure signals in the left ventricle, aorta and left atrium, f) the pressure signals in the right ventricle, pulmonary artery and right atrium, g) the volume signals in the left and right ventricles and atria, h) the ventricular and atrial diameters for the healthy child cardiovascular system.

First, the simulations were performed for the healthy adult and child cardiovascular system models. The ventricular, atrial and aortic pressures, the ventricular and atrial volumes and diameters in the healthy adult and child cardiovascular system models are given in Fig 2.

**Fig 3:**
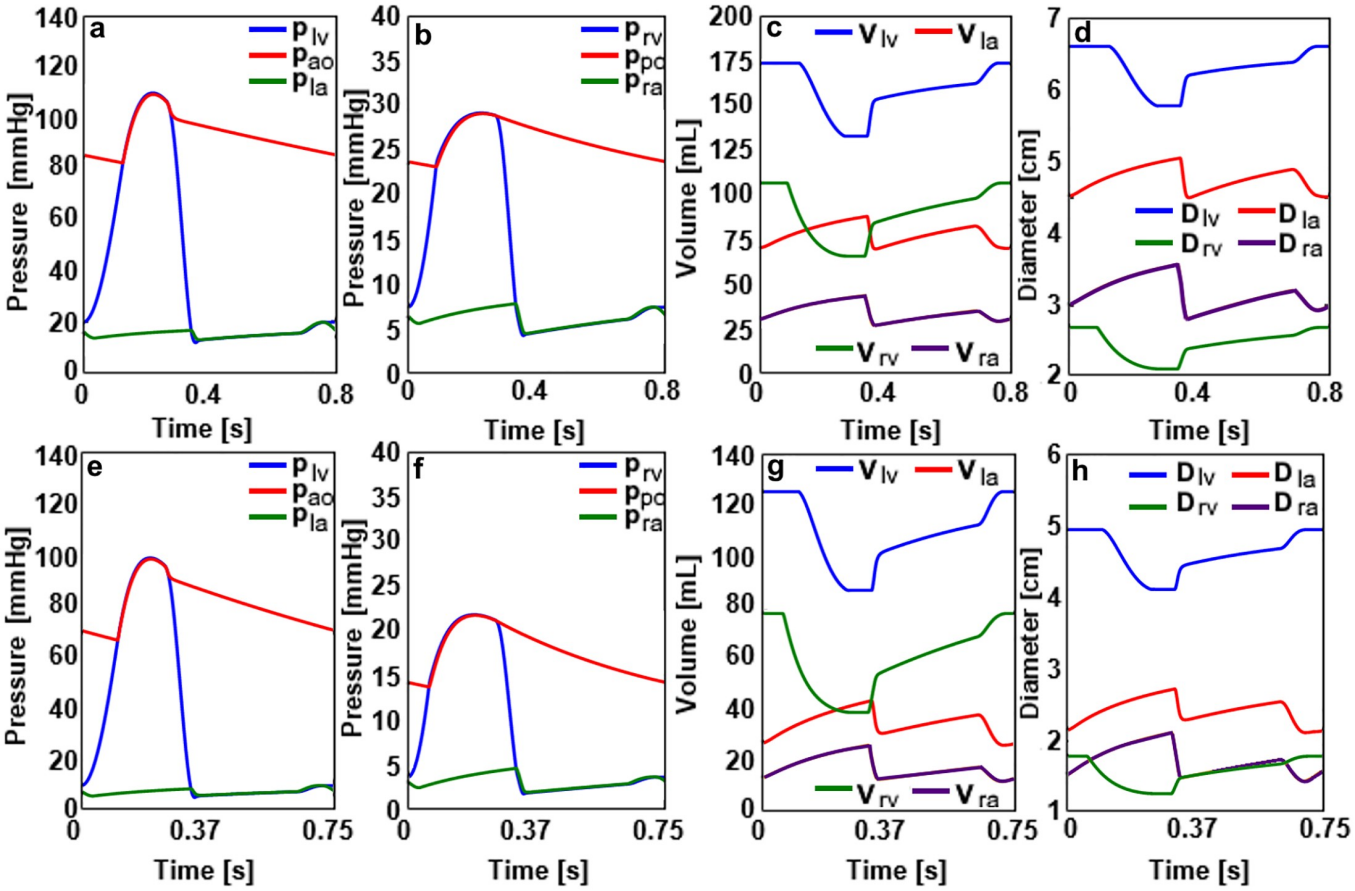
a) The pressure signals in the left ventricle, aorta and left atrium, b) the pressure signals in the right ventricle, pulmonary artery and right atrium, c) the volume signals in the left and right ventricles and atria, d) the ventricular and atrial diameters for the DCM adult cardiovascular system. e) The pressure signals in the left ventricle, aorta and left atrium, f) the pressure signals in the right ventricle, pulmonary artery and right atrium, g) the volume signals in the left and right ventricles and atria, h) the ventricular and atrial diameters for the DCM child cardiovascular system.

The left ventricular and aortic systolic pressures in the healthy adult cardiovascular system were around 120 mmHg. In both adult and child cardiovascular system models, the right ventricular volumes remain higher with respect to the left ventricular volumes over a cardiac cycle. The right atrial volumes are higher with respect to the left atrial volumes over a cardiac cycle in both models. Similarly, the right atrial diameters are higher as well with respect to the left atrial diameters over a cardiac cycle. The right ventricular basal diameter remains lower with respect to the left ventricular basal diameter similar to the normal physiological conditions in the adults and children. The ventricular and atrial pressures, aortic pressure, ventricular and atrial volumes and the diameters in the DCM adult and child cardiovascular system models are given in Fig 3.

**Fig 4:**
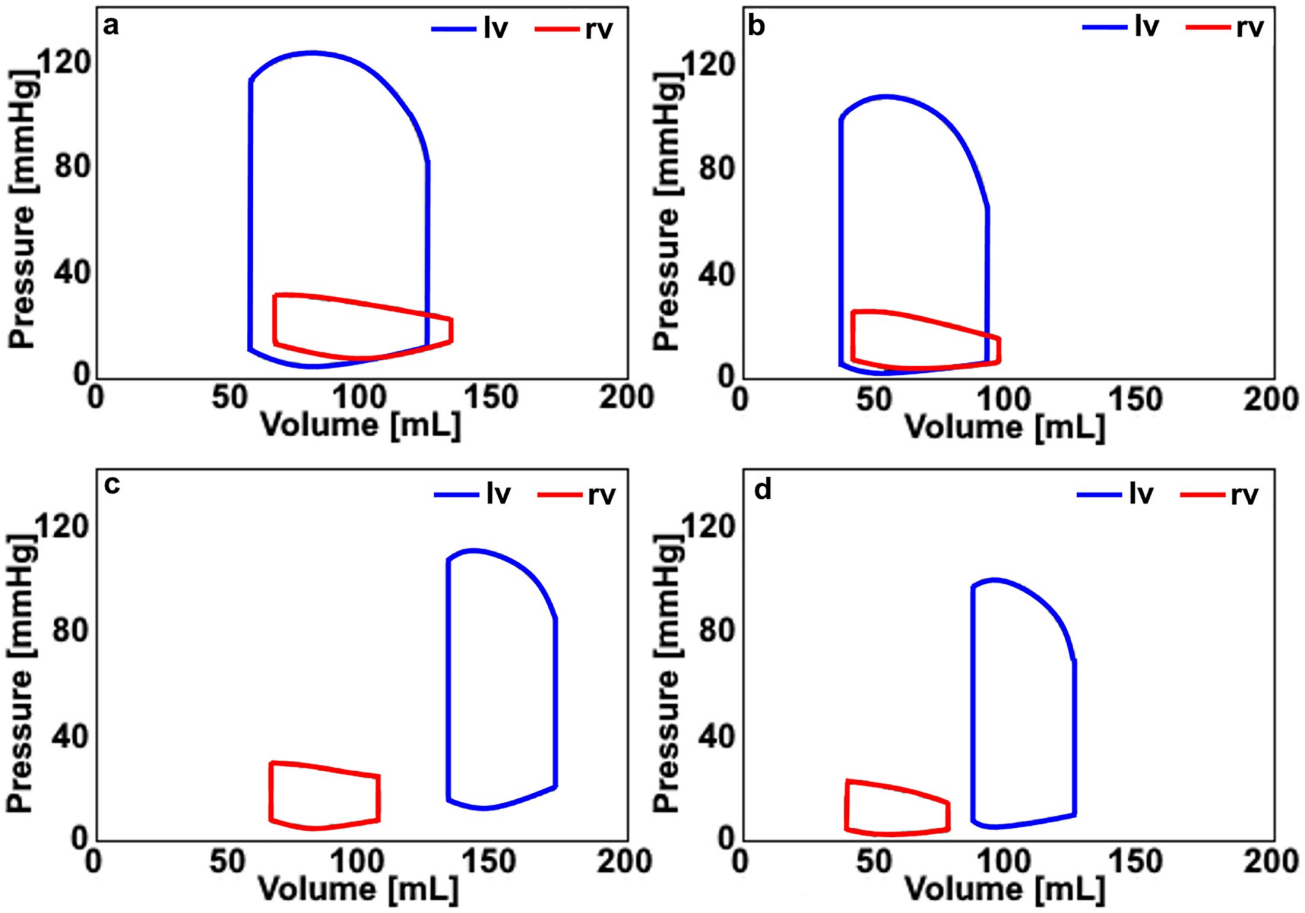
a) The pressure-volume loop signals in the left and right ventricles (lv, rv) for the healthy adult cardiovascular system model, b) the pressure-volume loop signals in the left and right ventricles (lv, rv) for the healthy child cardiovascular system model, c) the pressure-volume loop signals in the left and right ventricles (lv, rv) for the DCM adult cardiovascular system model, d) the pressure-volume loop signals in the left and right ventricles (lv, rv) for the DCM child cardiovascular system model.

The left ventricular and aortic systolic pressures decreased in the DCM cases for the adult and child cardiovascular system models. The left ventricular volume increased significantly simulating the enlargement because of the impaired contractility in the DCM left ventricles. Left atrial volumes in DCM models increased with respect to the left atrial volumes in the healthy cardiovascular system models. The left ventricular and atrial diameters increased as well with the increased volumes in the left atria and ventricles of the adult and child DCM cardiovascular system models. The pressure-volume loops for the healthy and DCM models are given in Fig 4.

**Fig 5:**
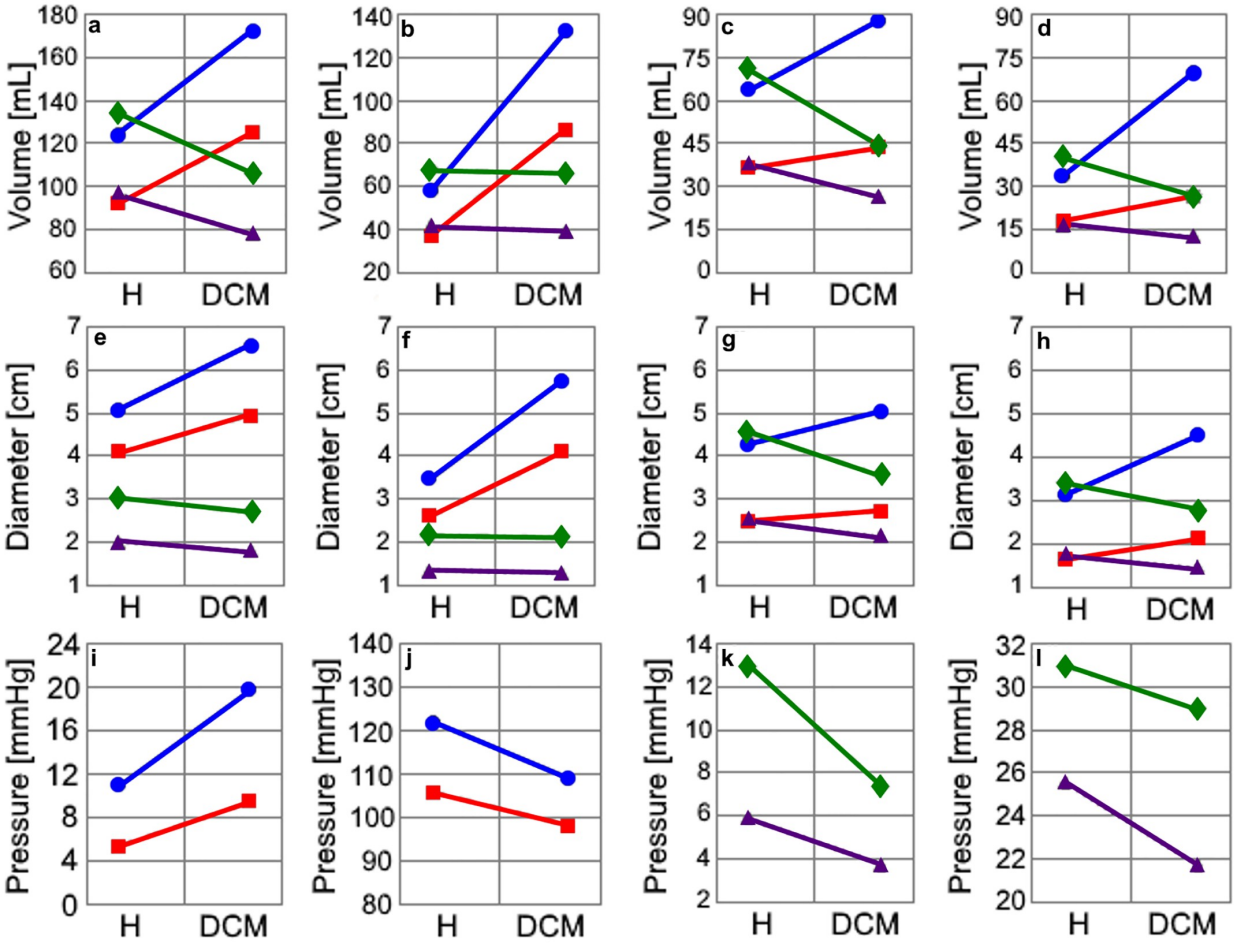
a) The left and right ventricular end-diastolic volumes, b) The left and right ventricular end-systolic volumes, c) The maximal left and right atrial volumes, d) The minimal left and right atrial volumes, e) The left and right ventricular end-diastolic diameters, f) The left and right ventricular end-systolic diameters, g) The maximal left and right atrial diameters, h) The minimal left and right atrial diameters, i) The left ventricular end-diastolic pressure, j) The left ventricular systolic pressure, k) The right ventricular end-diastolic pressure, l) The right ventricular systolic pressure. Hemodynamic parameters in the left heart of the adult circulation model (●), left the heart of the child circulation model (■), the right heart of the adult circulation model (♦), the right heart of the child circulation model (▲) for the healthy (H) and DCM conditions.

The left ventricular pressure-volume loop shifted to the right and became smaller in the cardiovascular system models simulating DCM for both adults and children. The pressure-volume areas of the left and right ventricles are smaller in the cardiovascular system models simulating the DCM condition with respect to the cardiovascular system models simulating the healthy condition. Additionally, the pressure-volume loop area is smaller in the child cardiovascular system model with respect to the adult cardiovascular system model. The end-systolic and end-diastolic volumes, diameters and pressures in the heart chambers for the adult and child cardiovascular systems with the healthy and DCM conditions are given in Fig 5, the maximal and minimal dimensions of the heart chambers and the hemodynamic parameters used to evaluate the healthy and DCM conditions are given in Table 5.

**Table 5:** The hemodynamic parameters used to evaluate the healthy and DCM conditions in the numerical model. LVEDD, LVESD, RVEDD and RVESD are left and right ventricular diameters at the end of diastole and systole, LAD_max_, LAD_min_, RAD_max_, RAD_min_ are maximal and minimal diameters in the left and right atria, CO and LVSV represent cardiac output and left ventricular stroke volume, LVEF and RVEF represent left and right ventricular ejection fractions, fs, Dsi and Ssi are fractional shortening in the left ventricular diameter, left ventricular diastolic and systolic sphericity indices, MAP and MPAP are the mean arterial and mean pulmonary arterial pressures.

	Healthy Adult CVS	DCM Adult CVS	Healthy Child CVS	DCM Child CVS
LVEDD [cm]	5.08	6.60	4.10	4.94
LVESD [cm]	3.48	5.77	2.60	4.11
RVEDD [cm]	3.02	2.70	2.01	1.79
RVESD [cm]	2.15	2.12	1.32	1.27
LAD_max_ [cm]	4.30	5.04	2.48	2.72
LAD_min_ [cm]	3.12	4.50	1.64	2.12
RAD_max_ [cm]	4.54	3.56	2.52	2.12
RAD_min_ [cm]	3.40	2.82	1.70	1.44
CO [L/min]	4.98	3.04	4.41	3.07
LVSV [mL]	66.4	40.6	55.1	38.4
LVEF [%]	53.6	23.7	59.7	30.7
RVEF [%]	49.63	37.74	56.94	49.46
fs [%]	31.5	12.5	36.6	16.8
Dsi	1.82	1.40	2.26	1.87
Ssi	2.65	1.60	3.54	2.26
MAP [mmHg]	97.8	92.4	81.8	80
MPAP [mmHg]	26.5	26	19.85	17.66

The hemodynamic parameters shown in Fig 5 and Table 5 were within the normal physiological ranges for the healthy and DCM condition in the adult and child cardiovascular system models. The left ventricular end-diastolic and end-systolic diameters increased in the DCM cardiovascular system models with respect to the healthy cardiovascular system models for an adult and a child. Left atrial maximal and minimal diameters increased in the DCM cardiovascular system models as well. Right ventricular end-diastolic diameters reduced while end-systolic diameters in the right ventricle do not change significantly for the DCM adult and child cardiovascular system models. Right atrial diameters reduced at the end-systole and end-diastole in the DCM adult and child cardiovascular system models. The cardiac output reduced significantly in the DCM condition for both adult child cardiovascular system models as well as the left ventricular stroke volume and ejection fraction. Fractional shortening, diastolic and systolic sphericity indices and aortic mean pressure decreased in the DCM conditions as well. The mean pulmonary arterial pressure remained at similar levels in the DCM and healthy conditions for both adult and child cardiovascular system models.

## Discussion

Time-varying elastance models reveal only the relationship between the pressure and volume signals in the ventricles by lumping all the contraction dynamics in the elastance term over a cardiac cycle. These models have been widely used to evaluate clinical devices or clinical scenarios numerically despite their limited capability [30,40]. The cardiovascular system model proposed in this study describes the relationship between pressure and dimensions of the heart chambers. Also, systolic elastances were used to describe pressures in the ventricle models. Therefore, the proposed numerical model extends the capability of the existing relatively simple time-varying elastance models without losing information. Single fiber contraction models are driven by contraction of muscle fibers, and they simulate length and tension on the fibers, along with the pressure and volume relation in heart chambers. Although single fiber contraction models have not been used as widely as the time-varying elastance models, they have been utilized to evaluate medical devices numerically [7,41]. These models also have limited capabilities in simulating indicators for clinical scenarios as ejection fraction is the only clinical indicator can be obtained. Therefore, their capability also remains limited with respect to the developed cardiovascular system model in this study. Multiscale models simulating the action potentials and Calcium kinetics provide detailed information about the cardiac dynamics at cellular and protein levels for a healthy and DCM heart [11]. However, at the organ level ejection fraction is the only parameter that such models can provide. Moreover, calcium dynamics in children at early ages are quite different [42], therefore, substantial modifications in the equations may be required to simulate heart failure when these models are used to simulate children cardiovascular system. The developed cardiovascular system model simulates cardiac function at organ level and allows simulation of additional clinical indicators such as sphericity index and fractional shortening, which are used in the diagnosis of heart failure. Moreover, the presented cardiovascular system model shows that children have a relatively long longitudinal axis with respect to the minor axis in their left ventricle.

Scaling coefficient (*K*) which include the effects of the longitudinal contraction and irregularities in the heart chamber geometries and allow simulation of the physiological heart chamber diameter signals was used in the proposed cardiovascular system models. The scaling coefficients (*K*) also relate the diameter and volume signals in the heart chambers. Modifying these parameters for a heart chamber does not influence the volume and pressure signals while the diameter changes. A higher value of the scaling coefficients (*K*) shows a stronger longitudinal contraction and a more complex geometry for the heart chambers. Right ventricle has the most complex geometry among all the heart chambers, and right ventricular longitudinal contraction contributes to the ejection of the blood through the pulmonary valve significantly [43]. Therefore, higher values for the right ventricular scaling constant (*K*_*rv*_) with respect to the scaling constants in other heart chambers were used in the adult and child cardiovascular system models. Also, higher scaling constant values for the heart chambers in the child cardiovascular system model with respect to the adult cardiovascular system show stronger longitudinal contractions in children. In the adult and child DCM models, the scaling coefficients (*K*_*lv*_) were modified as well as the systolic elastances (*E*_*sys*,*lv*_) and zero pressure-volume values (*V*_*lv*,*0*_) for the left ventricle because of the remodeling in the myocardial tissue causing in an increase in the left ventricular volume and diameter. The modifications in the systolic elastance (*E*_*sys*,*lv*_) and zero pressure-volume (*V*_*lv*,*0*_) were done to simulate the impaired contractile behavior and enlarged cavity volume in the DCM left ventricles. The scaling constant (*K*_*lv*_) was modified to simulate the changes in the contractile strength of the longitudinal contractions.

Ventricular long axis length in the model was assumed to be constant because the ventricular long axis signal over a cardiac cycle is similar and seems to be proportional to the ventricular volume signal [44]. A similar change in the ventricular diameter is also seen. Taking into account both signals increases the complexity of the model. Scaling coefficients (K) for each heart chambers were used to express the effects of longitudinal contraction and irregularities in the heart chamber geometries and reduce the complexity of the model.

Accuracy of the right ventricular volume estimation from 2D echocardiography requires assumptions on the geometry of right ventricle [45]. Trimmed ellipsoidal geometries similar to the presented right ventricular model in this study to estimate right ventricular volume have already been used in 2D echocardiography [46,47]. Empirical coefficients expressing the irregularities in right ventricular shape are also used in 2D echocardiographic area-length methods estimating right ventricular volume [45]. Using a trimmed ellipsoidal geometry and an additional coefficient (*K*_*rv*_) as in 2D echocardiographic cardiac imaging describes the right ventricular volume signals for the healthy and DCM models accurately.

The pressure and volume signals simulated in the adult cardiovascular system model for the healthy condition remain within the physiological range [27,48–51]. The simulated maximal and minimal heart chamber dimensions in the healthy adult cardiovascular system model are also within the healthy physiological ranges and corresponds to the reported average values for the adults [52–58]. As the dilatation occurs in a DCM left ventricle, the left ventricular volume increases significantly, internal ventricular diameter at the diastole and systole increase around 6.5 cm and 5.5 cm and ejection fraction may reduce below 30 per cent in adults [39,59,60]. The simulation results were in the reported physiological range for the adult DCM cardiovascular system model. Left ventricular geometry assumes a more spherical shape with the remodeling due to the dilation [61]. Reduced sphericity indexes at the end-systole and end-diastole in DCM (Table 5) indicate a more globular shape for the left ventricle [39]. Ejection fraction and fractional shortening are parameters used for diagnosis and risk stratification in DCM patients [39]. Moreover, fractional shortening is used for risk assessment in DCM [62]. Reduced ejection fraction and fractional shortening in the left ventricle (Table 5) indicate the existence of a dilated cardiomyopathy in the adult cardiovascular system model. [39]. The changes occur in the left ventricle also affect the other heart chambers and may increase the left atrial volume over a cardiac cycle or worsening the right ventricular function as in the simulations [63–65].

In pediatric patients, fractional shortening and ejection fraction are the most commonly used parameters to evaluate the left ventricular function [66]. Measurement of fractional shortening is based on the assumption of the cylindrical shape of the left ventricle. However, the assumption of cylindrical shape can be a limitation for using ejection fraction [66]. Relatively left ventricular high sphericity index values in the child cardiovascular system model (Table 5) indicate that the ratio between the long axis and mid-cavity diameter is relatively high. This indicates a close shape to a cylinder near the base of the left ventricle in the child cardiovascular system model. Children’s cardiac output reaches the similar levels to those in the adult cardiovascular system around 15 years while mean arterial pressure remains considerably lower in children at the same age with respect to mean arterial pressure level in adults’ cardiovascular system [36]. In this study, body surface area was selected as 1 m^2^ corresponding to 8–12 years children’s body surface area [31] and the hemodynamic parameters obtained from the simulations for the child cardiovascular system models compared to this age interval. The average cardiac output at these ages is around 4.5 L/min and changes within a large variation range [36]. The systolic pressure in children’s left ventricle generally remains below 110 mmHg while mean arterial pressure is around 80 mmHg in children circulation [67]. Reported right ventricular end-diastolic and systolic pressures are around 5 mmHg, 28 mmHg respectively and mean pulmonary arterial pressure can reach up to 22 mmHg being slightly lower with respect to those of adult cardiovascular system in the children around 9 years old [68]. The simulated pressures in the healthy child cardiovascular system were similar to those values reported in the literature. Mean of the left and right ventricular end-diastolic volume indexes in children are around 80 mL/m^2^ with slightly higher right ventricular volume and may reach to values around 100 mL/m^2^ while the end-systolic volume in the ventricles is around 30 mL/m^2^ and with 45 mL/m^2^ upper reference limit [51]. The volume indexes obtained from the simulations for 1 m^2^ body surface area corresponds well with the reported volume indexes in literature. The left atrial volume signal over a cardiac cycle in the healthy child cardiovascular system corresponds to a reported average left atrial volume signal over a cardiac cycle quite well [34]. Moreover, the reported range of the atrial maximal and minimal volume index values [51] matches with the simulated left and right atrial maximal and minimal volume indexes in the healthy child cardiovascular system for 1 m^2^ body surface area. Typically, the left ventricular end-diastolic and end-systolic diameters are around 4.1 cm and 2.6 cm, the right ventricular basal diameter at the end of the diastole is around 2 cm and the left atrial maximal diameter 2.9 cm and changing again within a physiologic variation range for the studied age interval [69–71]. The simulated dimensions in the cardiac chambers remain within the physiologic range and match with the reported values in the literature.

The DCM cardiovascular system model simulating the child circulation responded to the changes in parameters in a similar way as the adult DCM cardiovascular system model. Cardiac output and ejection fraction reduced from healthy values to the values characterized as impaired [72,73]. The left ventricular volume signal over a cardiac cycle increased as a result of dilatation and cause dilation in the left atrial volume, as reported in [74]. Left ventricular end-diastolic and end-systolic diameters and right ventricular diameter were similar to patient-specific values reported in [73] and remained within the reported range in the children for the studied age interval [75]. Moreover, the left ventricular fractional shortening in the child DCM cardiovascular system model matched closely to the reported average value of 19 per cent and remained within the physiologic variation range for the DCM condition in children heart [75].

Mitral regurgitation may occur in DCM because increased left ventricular size cause improper leaflet coaptation [38]. Simulation of the regurgitant flow rate through the mitral valve requires a numerical model describing the motion of leaflets [10,17] or zener diode analogue models allowing reverse blood flow. Flow rate signals through the heart valves were simulated using ideal diodes in the developed cardiovascular system model. Therefore, the change of the volume signals in the heart chambers depends only forward flow rate signals. Utilizing an elaborated mitral valve model will allow simulating effects regurgitant flow rate on the left ventricular function as the change of the left ventricular volume signal depends on mitral and aortic valve flow rates regardless of the direction of the blood flow (Eq 4). Nonetheless, ideal diode models have also been used widely to simulate clinical scenarios such as DCM and LVAD therapies in cardiovascular system [76,77].

The developed cardiovascular system model is driven by an activation function which utilizes time signal to simulate the hemodynamic variables. Clinically, wall stress and strain are also commonly used physiologic parameters. Simulation of these parameters requires performing finite element analyses since wall stress and strain have orientations in space and also depend on the geometry of myocardium. Additionally, the interaction between the ventricles through the intraventricular septum can be simulated using finite element analyses. The ventricles interact in the developed cardiovascular system model due to change in the parameters such as contractility as afterload and preload of the heart chambers are influenced because of variation of this parameter. However, it should be noted that the developed cardiovascular system model simulated the hemodynamic parameters for normal and DCM conditions in adults and children within the physiological range. Moreover, lumped parameter models have the advantage of performing simulations in shorter computational times, and they are powerful clinical decision-making tools [14].

The DCM in adult and child cardiovascular system models was simulated by adjusting the parameters in the cardiovascular system models manually. Progression of heart failure depends on complex and interacting mechanisms which are not entirely understood [78]. Modeling the progression of heart failure requires identification of these mechanisms. Therefore, progression of the DCM was not included in the simulations. Similarly, the interacting compensatory mechanisms in heart failure can be implemented after the identification of the exact mechanisms [79].

The cardiovascular system model presented in this study can be used to simulate different clinical cases for different ages and conditions as it allows to simulate clinical indicators extracted from the geometry of heat chambers. In the future, it is aimed to use this cardiovascular system model to simulate heart failure for pediatric patients in patient-specific studies to evaluate mechanical circulatory support. An important portion of the pediatric patients implanted with a heart pump exhibit complications associated with mechanical circulatory support [80]. Continuous and pulsatile flow LVADs are being tested to use in children. Moreover, LVAD success criteria in adults include evaluation of parameters such as ejection fraction, left ventricular end-diastolic volume, left ventricular end-diastolic diameter, left ventricular end-systolic diameter, left atrial volume, right atrial pressure [81]. The developed cardiovascular system model can simulate the dimensions of heart chambers along with the other parameters. The parameter values in the developed model can be tuned using parameter estimation and system identification techniques to simulate patient-specific cardiovascular system dynamics as presented in [4]. Therefore, it could be used to simulate patient-specific scenarios and evaluate devices such as LVADs before experimental tests.

## Conclusions

In this study, a mathematical model simulating hemodynamic parameters and clinical indicators in the cardiovascular system was developed to evaluate clinical cases in adults and children. The developed numerical model demonstrated its feasibility to simulate clinical cases and can be used to evaluate clinical cases and treatment techniques in adults and children at different ages.

## Supporting information

S1 File Cardiovascular system Model.slx, Matlab Simulink Code for model simulation. (ZIP)
